# Complete female-transmitted mitochondrial genomes of two freshwater mussels from the Lake Biwa system in Japan: *Nodularia douglasiae* and *N. nipponensis*

**DOI:** 10.1080/23802359.2021.1914218

**Published:** 2021-04-26

**Authors:** Kohji Mabuchi, Kazuya Nishida, Nobuyoshi Nakajima

**Affiliations:** a Lake Biwa Branch Office, National Institute for Environmental Studies, Otsu, Japan; b Environmental Genomics Office, National Institute for Environmental Studies, Tsukuba, Japan

**Keywords:** F mitogenome, *Nodularia douglasiae*, *Nodularia nipponensis*, *Nodularia douglasiae biwae*, Lake Biwa

## Abstract

We determined the complete mitochondrial sequences of female-transmitted (F) mitogenomes of two unionid specimens from the Lake Biwa system, Japan. Their gene contents and orders agreed with those of the typical F mitogenome of freshwater mussels. Molecular phylogenetic analysis using 20 previously identified partial COI and seven (five previously identified and two newly determined) whole mitogenome sequences revealed that one of the two mitogenomes was that of *Nodularia douglasiae*, while the other was *N*. *nipponensis*.

Freshwater mussels (Bivalvia: Unionidae) are burrowing, filter-feeding bivalves. They occur in lakes and rivers worldwide but are now declining in many countries (Carella et al. 2016). Conservation actions have been hindered by difficulty with species recognition and identification (Ferreira-Rodríguez et al. 2019) due to the high plasticity of shell shape within species and its convergence between species (Klishko et al. 2016). Recent molecular analyses are resolving the difficulties, and Lopes-Lima et al. (2020) recently proposed a new classification for Unionidae from Far East Asia, based on COI (cytochrome c oxidase subunit I) + 28S (28S ribosomal RNA) phylogenies. This new classification recognized two *Nodularia* species in Japan: *Nodularia douglasiae* (Gray, 1833) and *N. nipponensis* (Martens, 1877), the former occurring on Kyushu and Honshu Islands in the area along the Sea of Japan, and the latter being endemic to northern Honshu and Hokkaido. Before the new classification, however, two subspecies were recognized in Japan under the name, *Unio douglasiae* Gray, 1833: *Unio douglasiae nipponensis* Martens, 1877 and *Unio douglasiae biwae* Kobelt, 1879, with the latter endemic to the Omi Basin (Lake Biwa and adjacent area) on Honshu and the former occurring widely in Japan with the exception of the Omi Basin (Kondo 2008). The two subspecies were distinguished by the color of the glochidia: buff in the former and milky white in the latter (Kondo 1997).

Previously, we developed a DNA mini-barcoding system for unionids from the Lake Biwa system in Japan (Mabuchi and Nishida 2020). A single primer set was designed to amplify an approximately 140-bp barcode fragment within the mitochondrial 16S rRNA gene region, and reference DNA sequences for species identification were obtained using these primers. To adapt this barcoding system to the new classification, we determined the mitogenome sequences of two selected specimens called “*Nodularia douglasiae biwae*” in the barcoding system (specimen IDs B63 and n975 in Mabuchi and Nishida 2020). The two specimens were deposited in the Lake Biwa Museum, Shiga Prefecture, Japan (https://www.biwahaku.jp/, Masanari Matsuda, matsuda-masanari@biwahaku.jp), under the registration numbers LBM-1300014505 and 1300014533. Genomic DNA was isolated from foot muscle tissue, and sequenced using Illumina MiSeq and HiSeq X Ten sequencers (Illumina). The resultant reads were assembled using CLC Genomic Workbench (ver. 11.01; QIAGEN). Contigs were annotated by alignment with the two female (F)-transmitted mitogenomes of *N. douglasiae* (LC496352 and NC_026111) [Freshwater mussels are known to have male (M) and female (F)-transmitted mitogenomes showing different gene orders: Breton et al. 2009]. Using the two newly-determined mitogenomes, together with four additional ones of *N. douglasiae* (MT764726, LC496352, MF314443, and NC_026111) and 20 previously identified partial COI sequences of *N. douglasiae*, *N. nipponensis*, and *N. breviconcha* (Lee, Kim, Bogan and Kondo in Lopes-Lima et al. 2020) used in Lopes-Lima et al. (2020), a phylogenetic analysis was conducted to re-identify each of the two specimens, with the *Cuneopsis pisciculus* (Heude, 1874) mitogenome (KP273584) used as an outgroup. The phylogenetic tree was constructed using a supermatrix approach (de Queiroz and Gatesy 2007) as follows. The tree backbone was first generated for the seven mitogenomes by the neighbor-joining (NJ) method using the online version of MAFFT (https://mafft.cbrc.jp/alignment/server/). The obtained NJ tree was then used as a backbone constraint for the supermatrix tree, which was constructed based on the dataset including the seven mitogenomes and 20 partial sequences, which were first aligned using MAFFT and corrected by eye using Mesquite (version 3.31; http://www.mesquiteproject.org). After deleting the intergenic region, maximum likelihood analysis was performed for the resultant 14,803-bp dataset using RAxML BlackBox (https://embnet.vital-it.ch/raxml-bb/).

The resulting supermatrix tree (Figure 1) contained three major clades: *N. douglasiae*, *N. nipponensis*, and *N. breviconcha* [For the phylogenetic position of a “*N. douglasiae*” mitogenome (MT764726), detailed investigations including its taxonomic status will be needed as seen in Lopes-Lima et al. 2020]. The phylogenetic positions of the two mitogenomes indicated that one was that of *N. nipponensis* (together with the “*N. douglasiae*” mitogenome, LC496352), while the other was the new *N. douglasiae*. This result demonstrated that there are two mitogenome lineages of *Nodularia* in the Omi Basin, unlike the previous classification hypothesis of Kondo (2008), which recognized only a single taxon in the basin (the subspecies *U. douglasiae biwae*).

**Figure 1.**
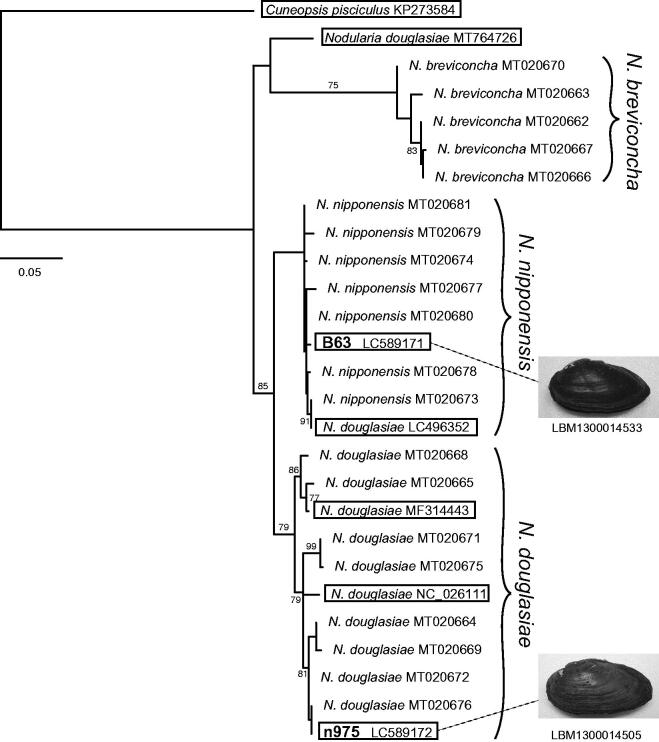
Supermatrix tree of seven mitogenomes (15,755–15,907 bp) and 20 partial COI sequences (612 bp) of female-transmitted (F) mitogenomes of *Nodularia* species (*Cuneopsis pisciculus* used as an outgroup). Bootstrap support (≥70%) is indicated at the nodes. For the previously identified sequences (20 partial sequences and five mitogenomes), accession numbers are given after the species names. For the two mitogenomes sequenced here, the accession numbers are indicated after the specimen IDs used in Mabuchi and Nishida (2020) (the IDs are in bold). The two newly sequenced and five published mitogenomes are boxed.

## Data Availability

Mitogenome data supporting this study are openly available in NCBI and DDBJ at nucleotide database, https://www.ncbi.nlm.nih.gov/nuccore/LC589171 and LC589172, Associated BioProjects, https://www.ncbi.nlm.nih.gov/bioproject/PRJDB11042 and PRJDB11041, BioSample accession numbers at https://www.ncbi.nlm.nih.gov/biosample/SAMD00271498 and SAMD00271497, and Sequence Read Archives at https://ddbj.nig.ac.jp/DRASearch/submission?acc=DRA011413, DRA011414, DRA011411, and DRA011412.

## Geolocation information

35.026259N, 135.869717E (B63); 35.446339N, 136.051366E (n975).
